# Author Correction: Probing the crystallographic orientation of two-dimensional atomic crystals with supramolecular self-assembly

**DOI:** 10.1038/s41467-018-08077-x

**Published:** 2019-01-08

**Authors:** Jinghui Wang, Hongde Yu, Xu Zhou, Xiaozhi Liu, Renjie Zhang, Zhixing Lu, Jingying Zheng, Lin Gu, Kaihui Liu, Dong Wang, Liying Jiao

**Affiliations:** 1 Key Laboratory of Organic Optoelectronics and Molecular Engineering of the Ministry of Education, Department of Chemistry, Tsinghua University, Beijing 100084, China; 2 State Key Laboratory for Mesoscopic Physics, Collaborative Innovation Center of Quantum Matter, School of Physics, Academy for Advanced Interdisciplinary Studies, Center for Nanochemisty, Peking University, Beijing 100871, China; 3 Beijing National Laboratory for Condensed Matter Physics, Institute of Physics, Chinese Academy of Sciences, Beijing 100190, China; 4 Key Laboratory of Colloid and Interface Chemistry of the Ministry of Education, Shandong University, Jinan 250100, China

Correction to: *Nature Communications* 10.1038/s41467-017-00329-6; published online: 29 August 2017

The original version of this Article omitted the following from the Acknowledgements:

‘Beijing Municipal Science & Technology Commission (No Z161100002116030)’

This has now been corrected in both the PDF and HTML versions of the Article.

