# Supplementary material for: Probing the crystallographic orientation of two-dimensional atomic crystals with supramolecular self-assembly
Source: Nat Commun. 2017 Aug 29;8:377. doi: 10.1038/s41467-017-00329-6 (PMC5575328; doi:10.1038/s41467-017-00329-6)
Supplement: Supplementary file 1 — Supplementary Information [file 41467_2017_329_MOESM1_ESM.pdf]

File Name: Supplementary Information
Description: Supplementary Figures, Supplementary Notes, Supplementary Methods and Supplementary References

File Name: Peer Review File
Description:

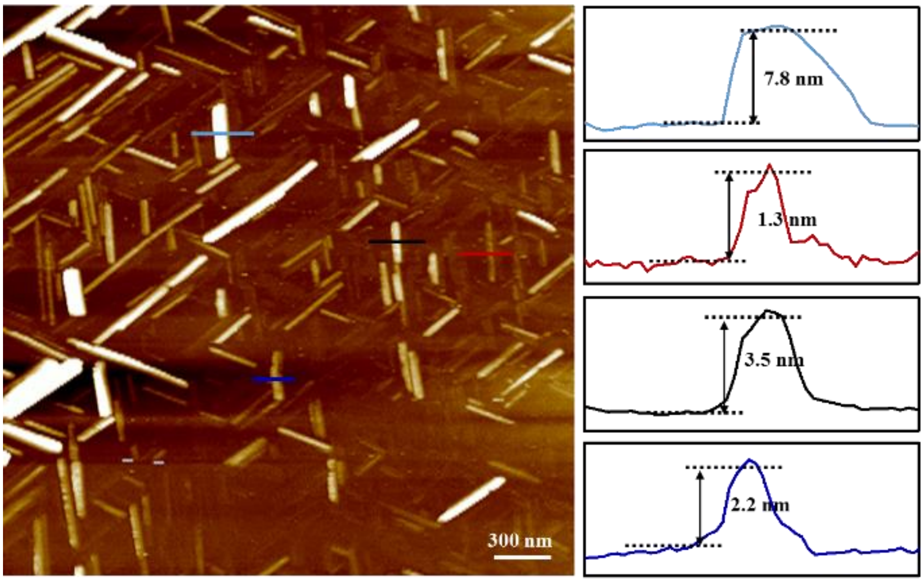

Supplementary Figure 1. Height analysis of several oleamide nanoribbons.

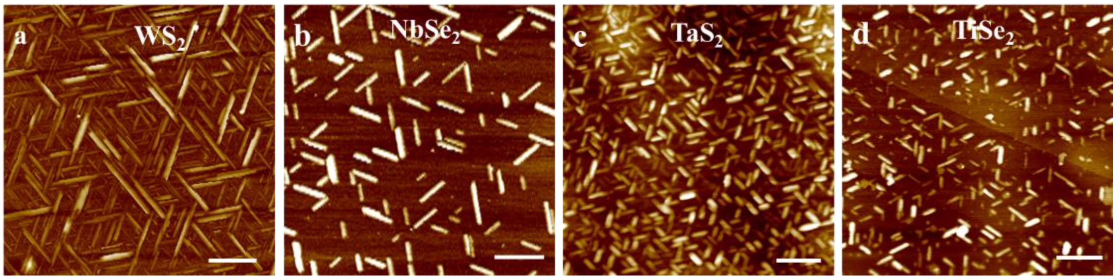

Supplementary Figure 2. AFM images of oleamide nanoribbons on WS_2 (a), $NbSe_2$ (b), TaS_2 (c) and $TiSe_2$ (d), also showing a three-fold symmetry. Scale bars: 500 nm.

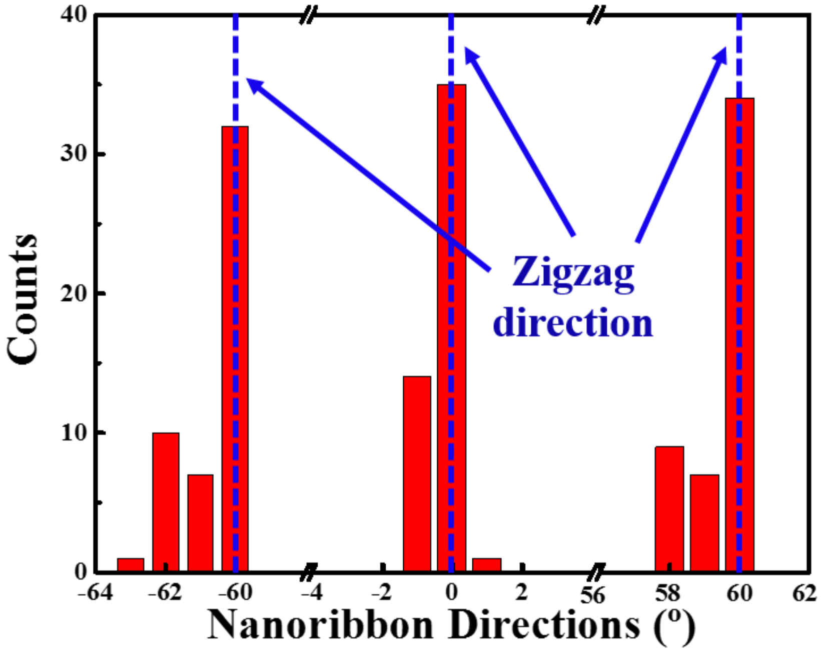

Supplementary Figure 3. Distributions of the orientations of nanoribbons on MoS_2 shown in Fig. 2a. Zigzag orientations were determined by STEM imaging.

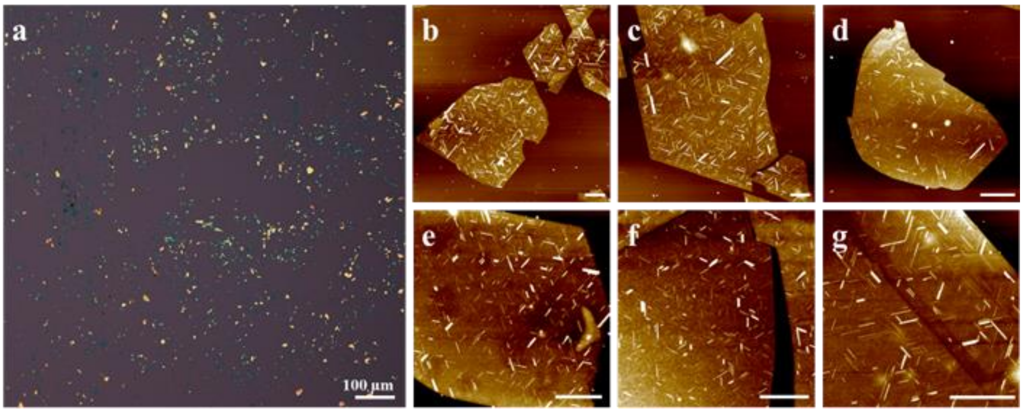

Supplementary Figure 4. The coverage of oleamide self-assembly on mechanically exfoliated MoS_2 flakes. **a,** The optical image of mechanically exfoliated MoS_2 flakes at a large scale. **b-g,** The AFM images of oleamide nanoribbons on MoS_2 flakes randomly selected at different locations on **a**. Scale bars: 1 μm.

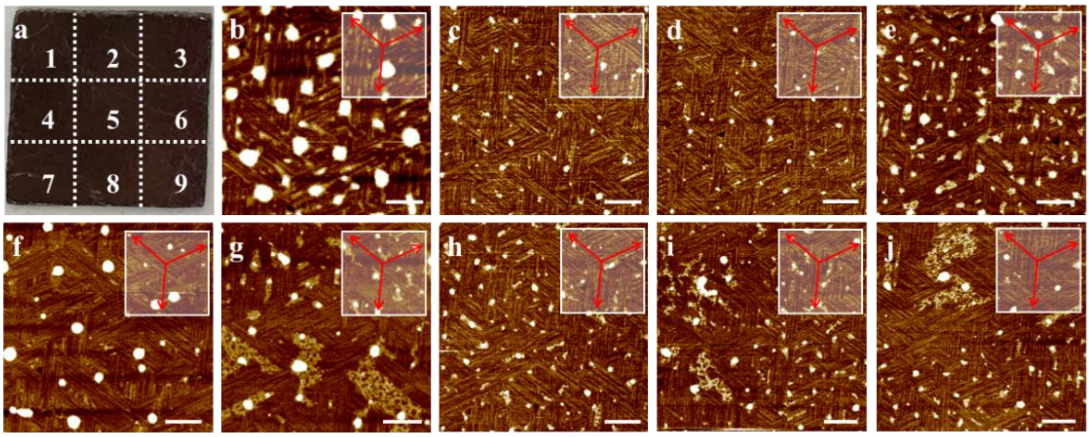

Supplementary Figure 5. The coverage of oleamide self-assembly on HOPG surface. **a,** The photo of a piece of 1 cm × 1 cm HOPG substrate which is divided into 9 regions. **b-j,** AFM images of oleamide self-assembly formed on the HOPG surface in the regions marked with 1-9. Scale bars: 300 nm.

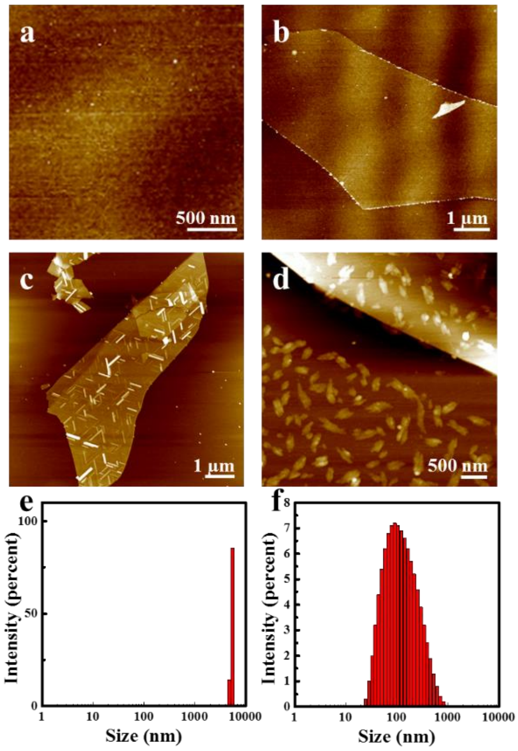

Supplementary Figure 6. Comparison between the self-assembly of oleamide and CoTPP on MoS_2 under the same experimental conditions. The self-assembly of both oleamide and CoTPP was conducted by spin-coating 1.65 mmol L^{-1} of oleamide or CoTPP in chloroform on MoS_2 at 2400 rpm for 1 min, followed by baking at 60 ^oC for 30 min . **a,** AFM image of bare SiO_2/Si. **b,** AFM image of a MoS_2 flake on SiO_2/Si. **c,** AFM image of oleamide assemblies on SiO_2/Si with MoS_2. Aligned oleamide ribbons were obtained only on MoS_2 flakes and no ribbons were found on the bare substrate. **d,** AFM image of CoTPP assemblies on SiO_2/Si with MoS_2. Randomly distributed CoTPP assemblies formed on both MoS_2 flakes and the substrate. **e,** Particle size distributions in 1.65 mmol L^{-1} solution of oleamide in chloroform measured by DLS. No particles with size of ~10-1000 nm in solutions was observed except for large particles (~10 μm) of impurities from the solvent. **f,** Particle size distributions in 1.65 mmol L^{-1} solution of CoTPP in chloroform measured by DLS. Particles with an averaged diameter of ~90 nm were well dispersed in the CoTTP solution. By combining the AFM imaging and DLS measurements, we can conclude that the nanoribbons of oleamide were formed on the surface of 2D materials instead of in solution.

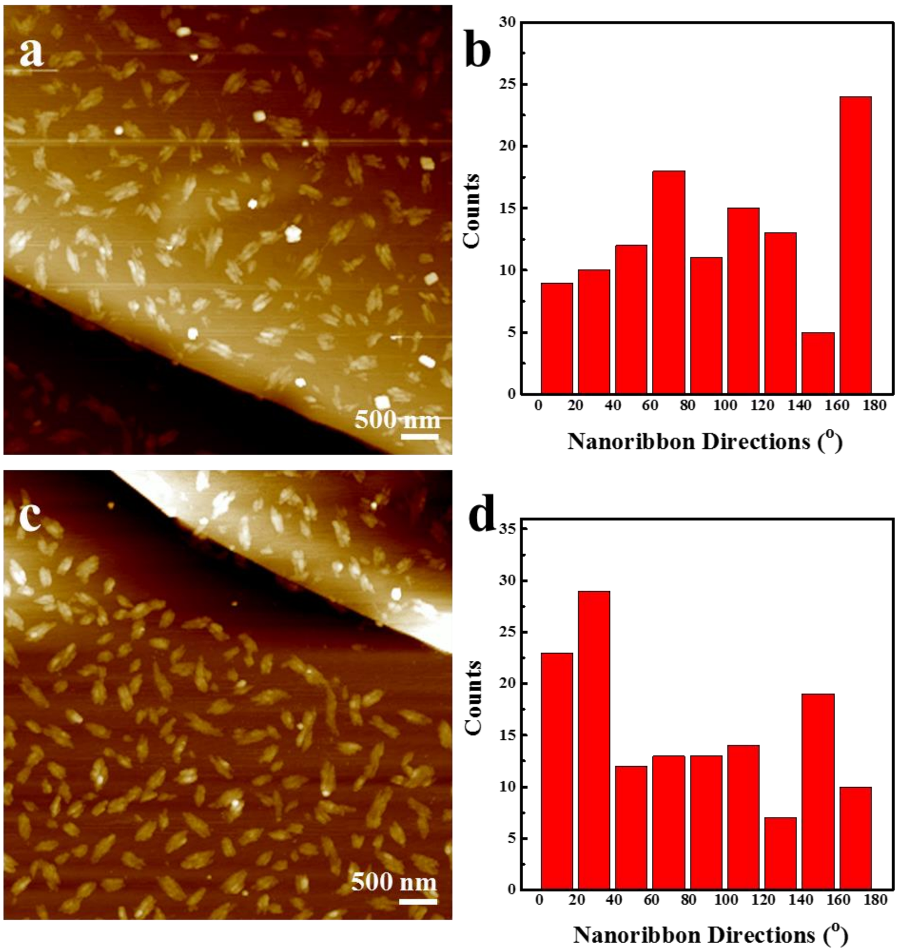

Supplementary Figure 7. Statistics of the orientations of CoTPP assemblies on MoS_2 and SiO_2/Si. **a,b** AFM image and histogram of the orientations CoTPP assemblies on MoS_2. **c,d** AFM image and histogram of the orientations of CoTTP assemblies on SiO_2/Si. These histograms suggested that CoTPP assemblies aligned randomly on both MoS_2 and SiO_2/Si. And by combing the DLS data shown in Supplementary Fig. 6f, we can infer that the assemblies of CoTPP were formed in solution.

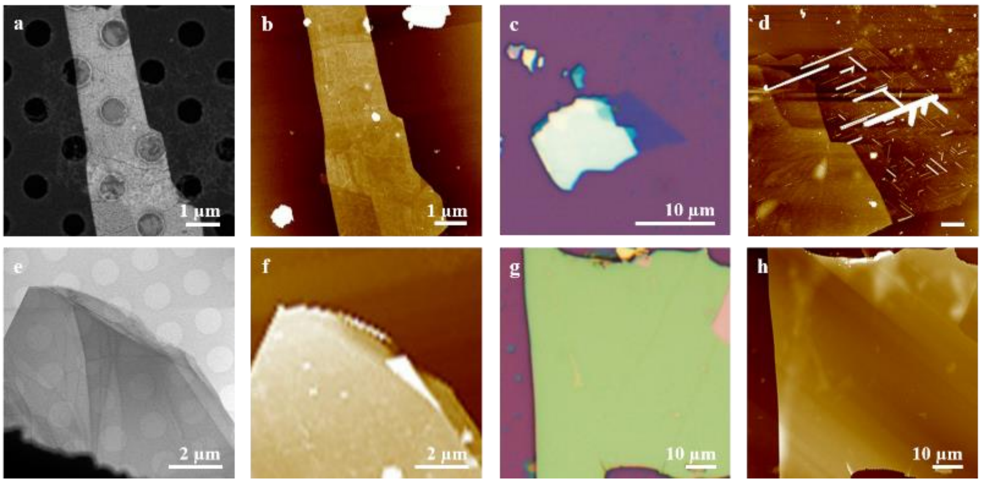

Supplementary Figure 8. Large-scale images of MoS_2 and black phosphorus flakes shown in Figure 2. **a,b,** TEM and AFM images of few-layer MoS_2 for STEM shown in Fig. 2a,b. **c,d,** Optical and AFM images of monolayer MoS_2 for SHG shown in Fig. 2c,d. **e,f,** TEM and AFM images of multi-layer black phosphorus for STEM shown in Fig. 2e,f. **g,h,** Optical and AFM images of multi-layer black phosphorus for polarized Raman shown in Fig. 2g,h.

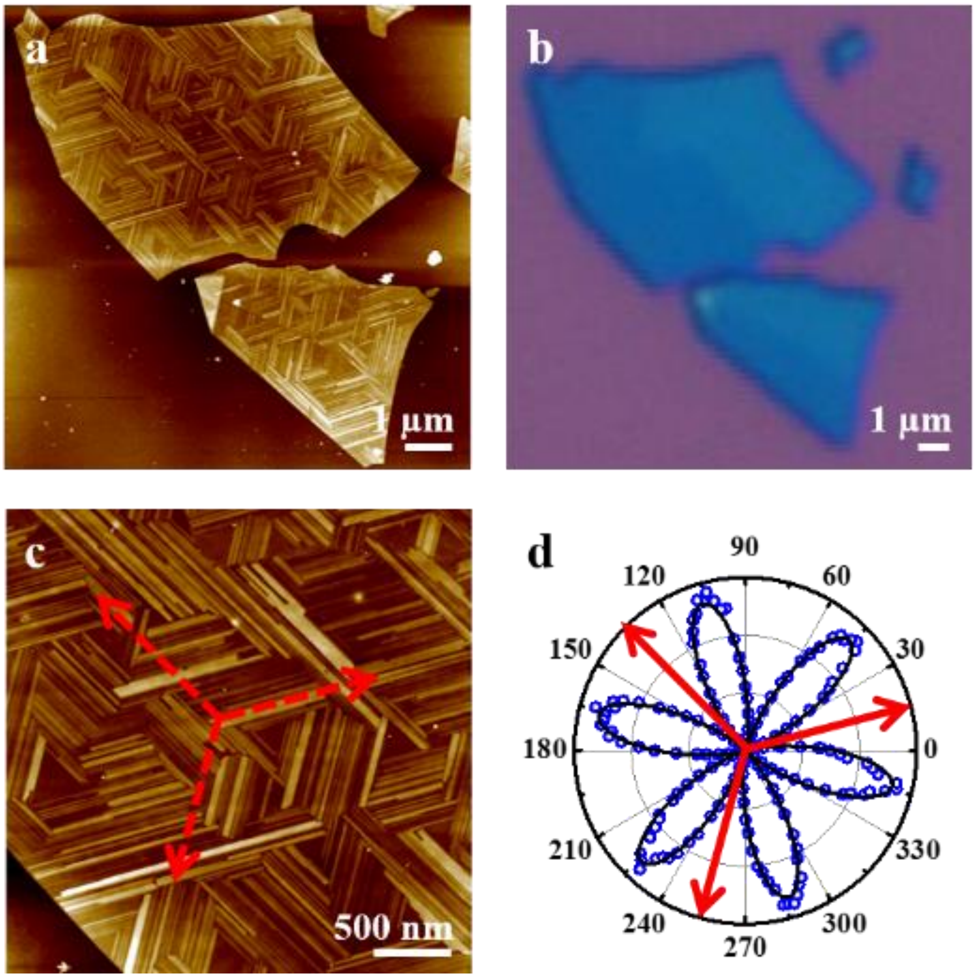

Supplementary Figure 9. Correlate the orientations of nanoribbons with the lattice of 2D WSe_2 by SHG. **a,b,** AFM and optical images of a few-layer WSe_2 flake for SHG measurement. **c,** Enlarged AFM image of oleamide nanoribbons on this WSe_2 flake. **d,** The polarization angle dependent SHG intensity of the same WSe_2 in **c**. Zigzag lattice orientations of WSe_2 are determined to be along red arrows as shown in **d**. The orientations of nanoribbons also match well with the zigzag directions of WSe_2.

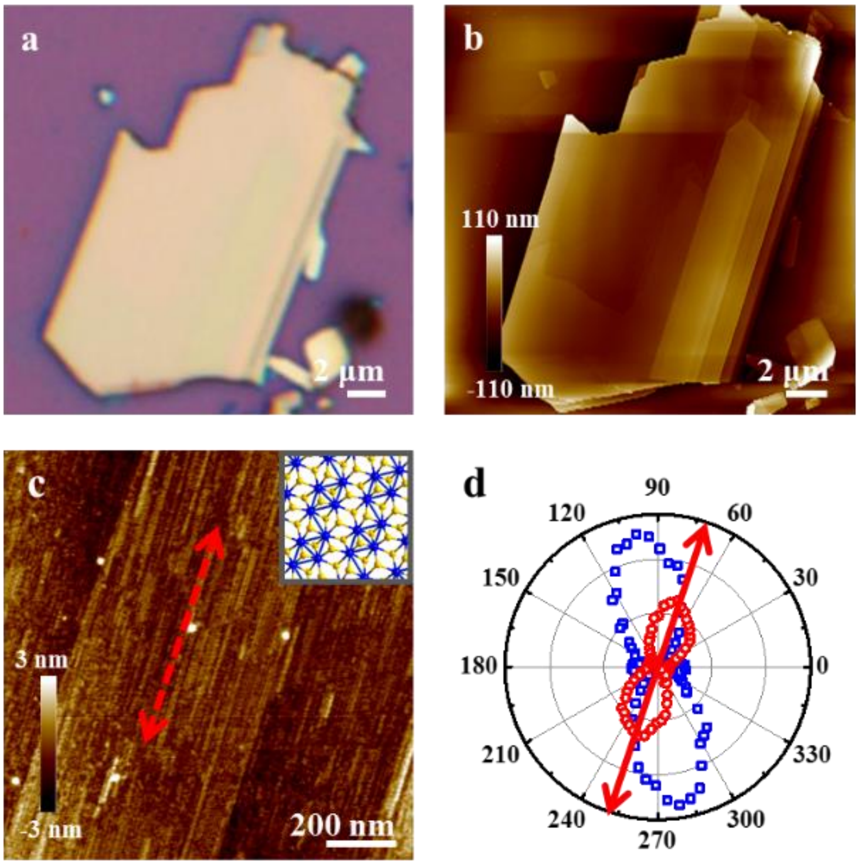

Supplementary Figure 10. Correlate the orientations of nanoribbons with the lattice of 2D ReS_2 by polarized Raman Spectroscope. **a,b,** AFM and optical images of a multi-layer ReS_2 flake for polarized Raman measurement. **c,** Enlarged AFM image of oleamide nanoribbons on this ReS_2 flake. **d,** Angle-resolved polarized Raman spectroscopy of the same multi-layer ReS_2 flake as shown in **c**. Zigzag lattice orientation of ReS_2 is determined to be along the red arrows as shown in **d**, indicating the orientation of oleamide nanoribbons is parallel to the Re chains in ReS_2 when the pre-annealing temperature is in the range of 290-300 ^{o}C.

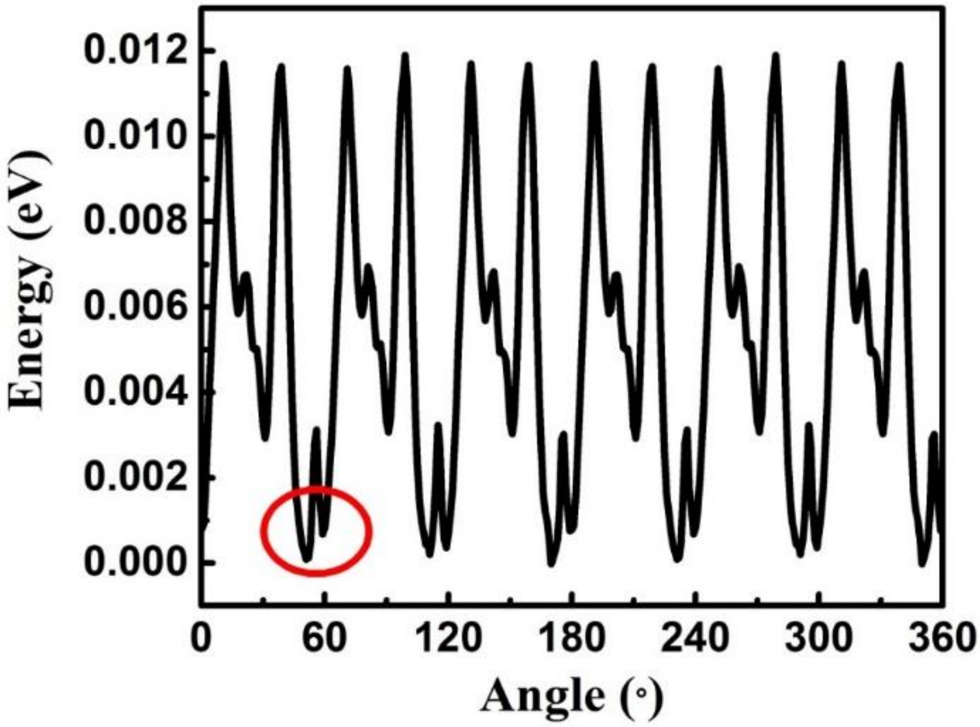

Supplementary Figure 11. Rotation potential energy curve of a single oleamide molecule on monolayer MoS_2. The two circled minimum values with the lowest energy represent the alignment of one of oleamide subchains along the zigzag lattice direction of MoS_2.

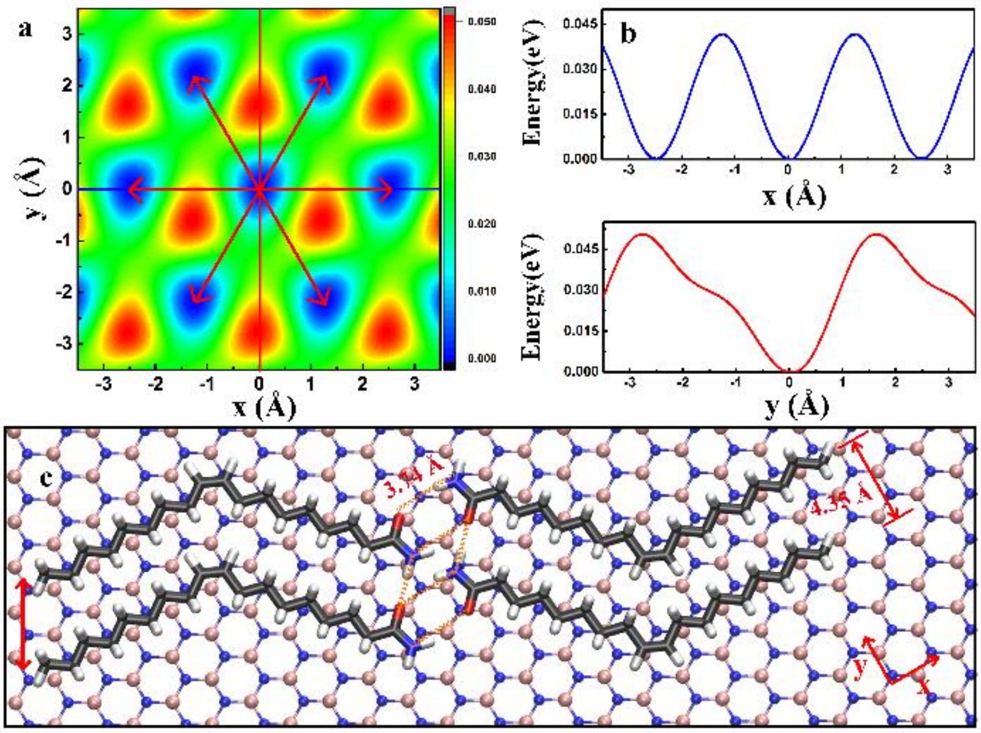

Supplementary Figure 12. Theoretical calculations of oleamide assembly on monolayer BN. **a,** 2D PES of a single oleamide molecule adsorbed on BN, with one of oleamide subchains aligning along the zigzag lattice direction of BN. **b,** 1D projection along the *x* (zigzag) and *y* (armchair) directions of the same 2D PES in **a**. **c,** Assembly of oleamide on BN. Each oleamide molecule is adsorbed onto the BN surface at the thermodynamically most stable position, as indicated by the energy valley of the 2D PES in **a**. In this assembly, oleamide molecules not only form H-bonded dimers in a head-to-head configuration, but also H-bonded nanoribbons in a side-by-side configuration. The red arrow on the left indicates the orientation of the nanoribbon, which is exactly the zigzag lattice directions of BN. The color code of the atoms is B: pink, N: blue, C: grey, H: white, and O: red respectively.

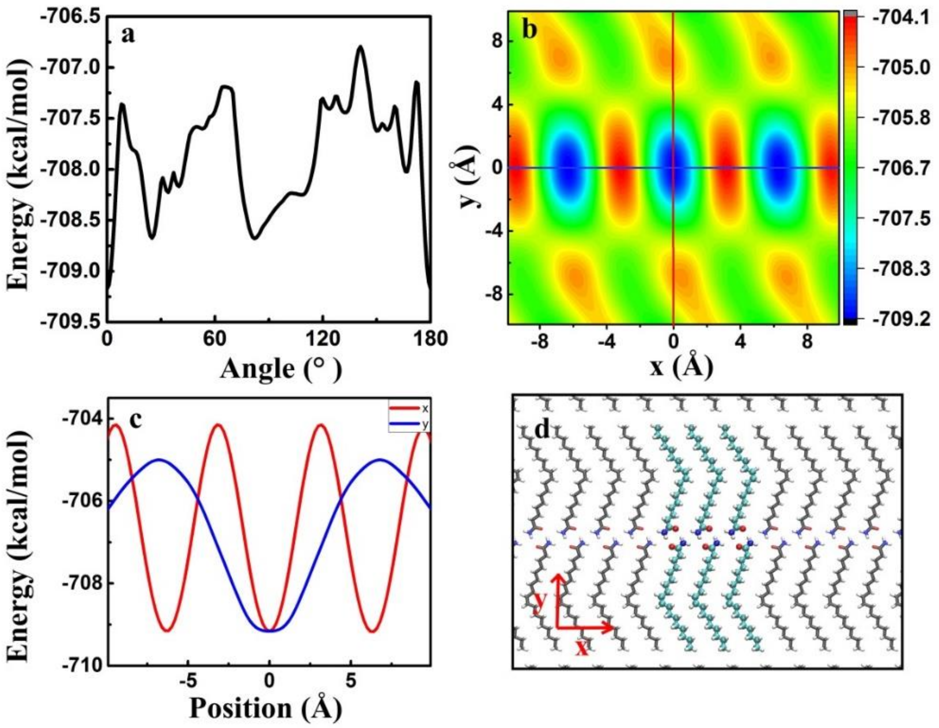

Supplementary Figure 13. Theoretical calculations of the 2nd layer oleamide assembly on the 1st layer of oleamide nanoribbon. **a,** Rotation potential energy curve of an oleamide dimer on top of the single-layered oleamide nanoribbon. The minima at 0° and 180° indicates that the 2nd layer oleamide aligns parallel to the 1st layer ones, and along the zigzag lattice direction of the substrate. **b,** 2D translation PES of a zigzag aligned oleamide dimer adsorbed on the oleamide nanoribbon. The energy valleys in blue represent the most stable adsorption positions, at which the 2nd layer molecules stack right on top of the 1st layer ones. **c,** 1D projection along the *x* (zigzag) and *y* (armchair) directions of the same 2D PES in **b**. **d,** Assembly of the 2nd layer oleamide on the 1st layer of oleamide nanoribbon. Each oleamide dimer is adsorbed onto the nanoribbon at the thermodynamically most stable position, as indicated by the energy valley of the 2D PES in **b**. The color code of the atoms is C: cyan (2nd layer) and grey (1st layer), H: white, O: red, and N: dark blue, respectively.

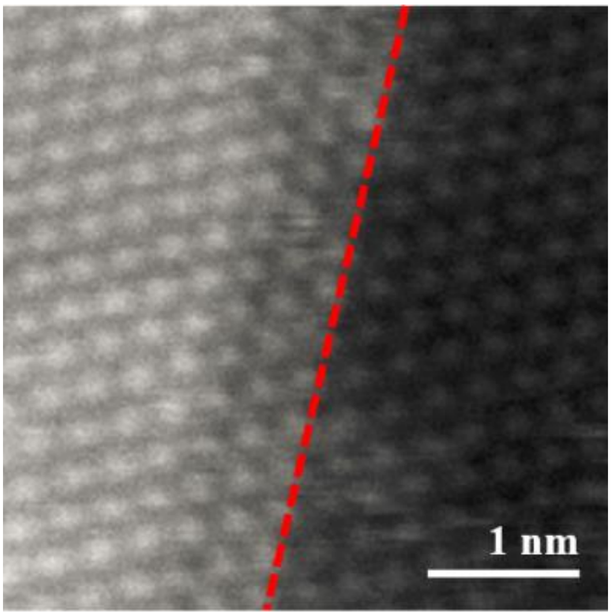

Supplementary Figure 14. STEM characterization of the edge structure of a CVD-grown multilayer MoS_2. The bright region is trilayer MoS_2 and the dark region is monolayer MoS_2. The red dashed line indicates the Mo-zigzag orientation.

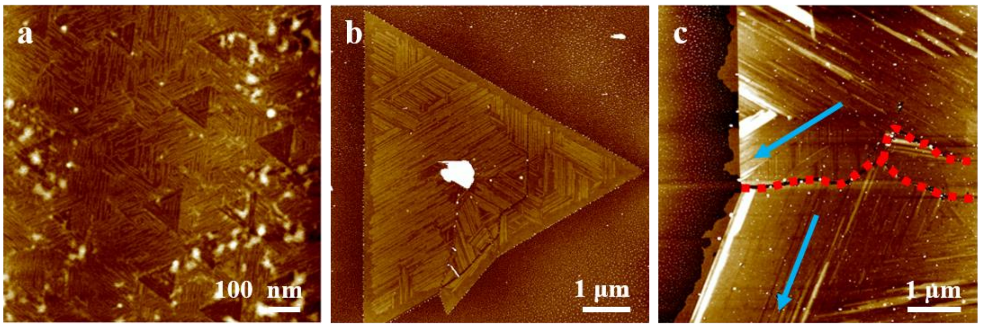

Supplementary Figure 15. a, AFM image of oleamide nanoribbons on MoS_2 with etched triangular pits. **b,c,** AFM images of oleamide nanoribbons on CVD-grown polycrystalline MoS_2. The grain boundaries were marked with red dotted lines.

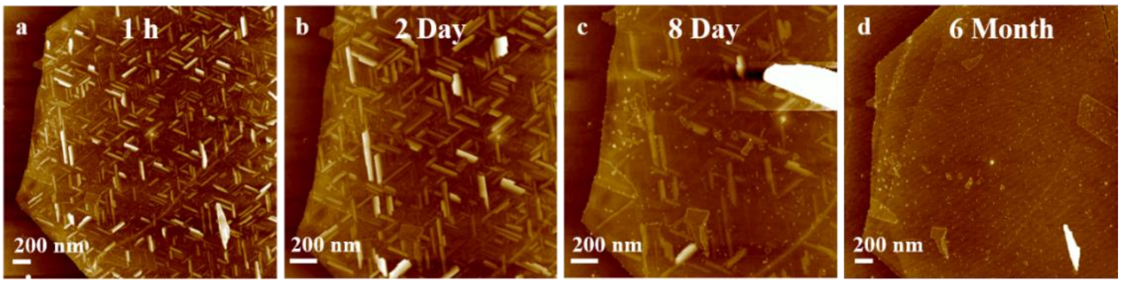

Supplementary Figure 16. Evolution of self-assembled nanoribbons on MoS_2 surface. **a-d,** AFM images of nanoribbons after storing under ambient condition for 1 h, 2 days, 8 days and 6 months, respectively.

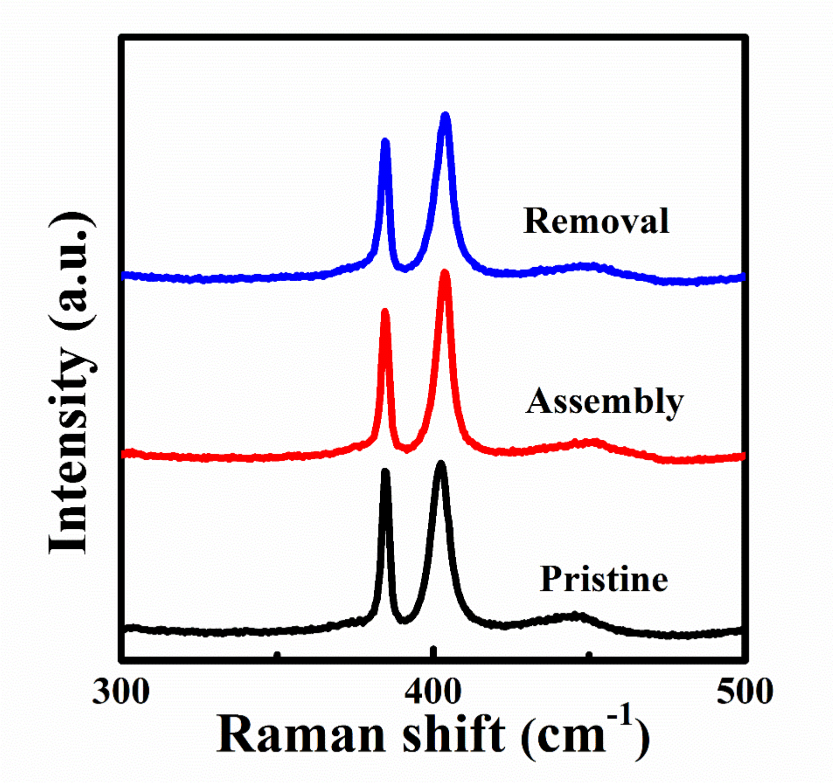

Supplementary Figure 17. Typical Raman spectra of pristine MoS_2 after the removal of residual tape (black), oleamide self-assembly (red) and the removal of nanoribbons (blue), respectively.

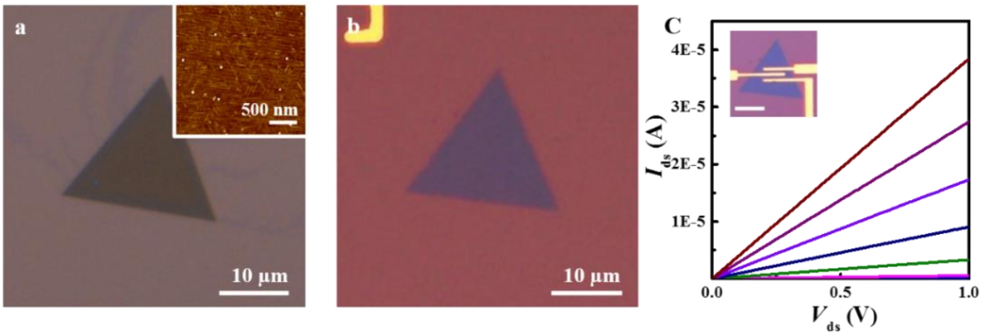

Supplementary Figure 18. Electrical properties of a typical FET made on MoS_2 after the assembly and removal of oleamide. **a,** Optical image of monolayer MoS_2 with the self-assembled nanoribbons. Self-assembly can be visualized under optical microscope. Inset: AFM image of nanoribbons. **b,** Optical image of the same MoS_2 after transfer and removal of self-assembly. **c,** I_{ds}-V_{ds} curves for a MoS_2 flake after the removal of self-assembly at varied V_{gs} from -40 V to 40 V at steps of 10 V from bottom to top. Inset: Optical microscope image of the device. Scale bar: 10 µm.

Supplementary Note 1. Correlation of the orientations of nanoribbons with the lattice structure of 2D atomic layers.

Taking Fig. 2a, 2b, Supplementary Fig. 8a, 8b as an example, the correlation between the orientations of nanoribbons and the MoS_2 atomic structures were determined by the following procedures: We first recorded the geometry of MoS_2 flakes with oleamide assembly (Supplementary Fig. 8b) and the orientations of oleamide (Fig. 2a) using AFM. Then we transferred the same MoS_2 flake to a holey carbon grid and imaged the geometry (Supplementary Fig. 8a) and atomic structure (Fig. 2b) of MoS_2 flake by STEM. Finally, we rotated the geometric image taken by AFM (Supplementary Fig. 8b) to exactly overlap with the STEM image (Supplementary Fig. 8a) and then rotated Fig. 2a to the same degree and matched with Fig. 2b to determine the relationship between the orientations of nanoribbons and the MoS_2 atomic structures.

Supplementary Note 2. Theoretical calculations of oleamide assembly on other hexagonal atomic crystals.

Besides MoS_2, we also calculated the assembly of oleamide on BN as shown in Supplementary Fig. 12 and the obtained results also confirmed that the oleamide nanoribbons are preferred to align along the zig-zag directions of underlying BN. The assembly of oleamide on other hexagonal atomic crystals is similar to that on monolayer MoS_2. The alignment of oleamide along the zigzag lattice directions of these atomic crystals is energetically favored as in the case of MoS_2. Importantly, the energy valleys exhibit the same hexagonal symmetry as the underlying hexagonal lattice of atomic crystals. To maximize intermolecular interactions of oleamide and facilitate the supramolecular assembly, the nanoribbons have to be formed in the zigzag lattice directions of the underlying hexagonal atomic crystals.

Supplementary Note 3. Theoretical calculations of the 2nd layer oleamide assembly on the 1st layer of oleamide nanoribbon.

To investigate the molecular orientation in the bulk of nanoribbon, we performed the potential energy scan of an oleamide dimer on the 1st molecular layer of nanoribbon with similar computational method described above. The rotation (Supplementary Fig.

13a) and translation (Supplementary Fig. 13b,c) potential energy curves show that oleamide dimer in the 2nd layer prefers to align parallel to the underneath 1st layer dimer and stacks right on top of it. Based on the thermodynamically most stable adsorption position of oleamide dimers, we constructed the assembly pattern of the 2nd oleamide layer on the 1st layer of nanoribbon, as shown in Supplementary Fig. 13d. In this pattern, the 2nd layer molecules assemble in the same way as the 1st layer ones. In other words, the surface lattice of atomic crystals templates the assembly of the 1st layer oleamide molecules, and the 1st layer of nanoribbon thus formed serves as template for the 2nd layer molecular assembly, and so on. Thus, the 2nd layer molecules and all layers above will align along the same direction as the 1st layer.

Supplementary Note 4. Oleamide nanoribbons on etched MoS_2 with triangular pits and CVD-grown polycrystalline MoS_2.

The etched triangular pits were obtained by annealing in vacuum at a pressure of 10 Pa at 660 ^{o}C for 20 min. The orientations of nanoribbons were parallel to the edges of pits, indicating that the etched edges are all along the zigzag directions, in agreement with previous reports[1]. Due to the correlation between nanoribbons and lattice structure of underlying MoS_2, different crystalline grains guide the molecular self-assemble into different orientations. Therefore, grain boundaries can be identified by the changed nanoribbon orientations (marked with blue arrows) as shown in Supplementary Fig. 15c.

Supplementary Note 5. Raman and PL spectra of MoS_2 after the removal of oleamide.

No obvious Raman shift was observed on >10 mechanical exfoliated monolayer MoS_2. In Figure 4f, 30% quench of PL intensity after self-assembly and small enhancement after removal were observed, which is likely to be attributed to the adsorption of oleamide and impurities under ambient condition[2].

Supplementary Methods

Transfer of MoS_2 flakes and CVD-grown MoS_2. A PMMA-mediated transfer approach was harnessed to transfer the mechanically exfoliated MoS_2 to a holy carbon TEM grids for STEM characterizations. Firstly, the SiO_2/Si substrate was spin coated with PMMA (Allresist GmbH, AR-P 679.04) at 3000 rpm for 1 min, followed by baking at 170 ^oC for 2 h. Then the substrate was immersed in KOH solution (5 wt%) to detach the PMMA film from the substrate. After rinsing thoroughly with deionized water, the PMMA film was attached to TEM grids. Finally, the PMMA film was removed by acetone vapor. A PVP-PVA-mediated transfer approach was utilized to transfer the CVD-grown MoS_2 to SiO_2/Si chips with markers for device fabrication[3]. Firstly, the SiO_2/Si substrate was spin coated with a PVP (Alfa Aesar, average M.W. 58,000) and NVP (J&K, 99.5%) ethanol solution (0.75 g PVP, 1.5 mL NVP and 0.75 mL H_2O dissolved in ethanol to prepare 10 mL solution) at 2500 rpm for 1 min, followed by baking at 70 ^oC for 1 min. Then 8.9 wt% PVA (Alfa Aesar, 98-99% hydrolyzed, high molecular weight) aqueous solution was spin-coated on the top of PVP film at 2500 rpm for 1 min and baked at 70 ^oC for 1 min. The polymer mediator was peeled off from the substrate after being scratched by a blade at the edges. Finally, the polymer film was attached to SiO_2/Si chips with markers and removed by socking in deionized water at 70~80 ^oC for 20 min.

Device fabrication and measurement. Source and drain electrodes (5 nm Ti/50 nm Au) were fabricated on monolayer MoS_2 flakes by electron-beam lithography followed by thermal evaporation of Ti and Au on 300 nm SiO_2/Si substrates[4]. The obtained FETs were measured with probe station under high vacuum (~10^{-6} mbar) at room temperature using Agilent B1500A. The mobility was calculated using the following equation[4]:

$$\mu = \frac{L}{W \times (\varepsilon_0 \varepsilon_r / d) \times V_{ds}} \times \frac{dI_{ds}}{dV_g} \quad (1)$$

Removal of nanoribbons. The nanoribbons on 2D materials were removed by annealing in vacuum at a pressure of 10 Pa at 300 ^oC for 1 h under a heating rate of 20 ^oC min^{-1}.

Potential energy surface scan. The oleamide molecule was adsorbed with the carbon backbone parallel to the surface of atomic crystals and at a fixed distance of 3.5 Å. The oleamide as a rigid body has three degrees of freedom to move on the surface: in-plane rotations and translations. The oleamide was rotated on the surface of atomic crystals at an interval of 1, and for each orientation of the oleamide molecule, a translation potential energy surface scan was performed with only Grimme's D3 dispersion parameters[5] to locate the most favorable adsorption position of the oleamide on the surface. During the scan, the oleamide was translated along the *x* (zigzag) and *y* (armchair) directions, respectively, at an interval of 0.1 Å. Since the surface of atomic crystals is periodic, the translation potential energy surface exhibits the same symmetry and periodicity as the underlying lattice of atomic crystals. The rotation potential energy curve was subsequently constructed based on the energy valleys obtained during the translation potential energy surface scans. It showed that the zigzag alignment of oleamide on the surface of hexagonal atomic crystals is energetically most favorable.

Adsorption energy calculation. The adsorption energy was calculated for the zigzag and armchair aligned oleamide, respectively at its most favorable adsorption position on MoS_2. The calculation of the adsorption energy was based on the density functional theory (DFT) and the projector augmented wave (PAW) method, as implemented in the Vienna ab initio simulation package (VASP, version 5.3.5)[6]. The Perdew−Burke−Ernzerhof (PBE) exchange-correlation functional with Grimme's dispersion correction (D3) was applied to account for the van der Waals interactions between oleamide and the surface of atomic crystals. The convergence criterion of the total energy was set to be 10^{-5} eV in the self-consistent field iteration. The cutoff energy for the plane-wave basis set was 600 eV and the spin-polarization was not considered. The cutoff radius for pair interactions was set to be 50 Å and the Monkhorst-Pack k-mesh of 1×1×1 was used. The substrate was constructed by a monolayer of atomic crystals, which was large enough to accommodate a single oleamide molecule and to avoid interactions between periodic images of oleamide. A vacuum of 30 Å was included in the out-of-plane direction.

Supplementary References

1. Yamamoto, M., Einstein, T. L., Fuhrer, M. S. & Cullen, W. G. Anisotropic etching of atomically thin MoS_2. *J. Phys. Chem. C* **117,** 25643–25649 (2013).
2. Oh, H. M. *et al.* Photochemical reaction in monolayer MoS_2 via correlated photoluminescence, raman spectroscopy and atomic force microscopy. *ACS Nano* **10,** 5230–5236 (2016).
3. Lu, Z. *et al.* Universal transfer and stacking of chemical vapor deposition grown two-dimensional atomic layers with water-soluble polymer mediator. *ACS Nano* **10,** 5237–5242 (2016).
4. Wang, X., Feng, H., Wu, Y. & Jiao, L. Controlled synthesis of highly crystalline MoS_2 flakes by chemical vapor deposition. *J. Am. Chem. Soc.* **135,** 5304–5307 (2013).
5. Grimme, S., Antony, J., Ehrlich, S. & Krieg, H. A consistent and accurate ab initio parametrization of density functional dispersion correction (DFT-D) for the 94 elements H-Pu. *J. Chem. Phys.* **132,** 154104 (2010).
6. Kresse, G. & Furthmüller, J. Efficient iterative schemes for ab initio total-energy calculations using a plane-wave basis set. *Phys. Rev. B* **54,** 11169–11186 (1996).
